# Chromosome-level genome assembly of *Fragaria pentaphylla* using PacBio and Hi-C technologies

**DOI:** 10.3389/fgene.2022.873711

**Published:** 2022-09-06

**Authors:** Rui Sun, Shuangtao Li, Linlin Chang, Jing Dong, Chuanfei Zhong, Hongli Zhang, Lingzhi Wei, Yongshun Gao, Guixia Wang, Yuntao Zhang, Jian Sun

**Affiliations:** ^1^ Institute of Forestry and Pomology, Beijing Academy of Agriculture and Forestry Sciences, Beijing, China; ^2^ Beijing Engineering Research Center for Strawberry, Beijing, China; ^3^ Key Laboratory of Biology and Genetic Improvement of Horticultural Crops (North China), Ministry of Agriculture, Beijing, China

**Keywords:** chromosomal assembly, *Fragaria pentaphylla*, gene annotation, Hi-C, PacBio

## Abstract

*Fragaria pentaphylla*, a wild diploid quinquefoliolate species of *Fragaria*, is native to Southwest China. It has two morphs of red and white fruit color in nature and has characteristics of unique fragrance and resistance, which made it not only a valuable breeding material but also a potential model plant for molecular function researches. Here, we generate a high-quality chromosome-level genome assembly of a *F. pentaphylla* accession, BAAFS-FP039 employing a combination of PacBio Long-Read Sequencing, Illumina Short-Read Sequencing, and Hi-C Sequencing. The assembled genome contained 256.74 Mb and a contig N50 length of 32.38 Mb, accounting for 99.9% of the estimated genome (256.77 Mb). Based on Hi-C data, seven pseudo-chromosomes of *F. pentaphylla*-FP039 genome were assembled, covering 99.39% of the genome assembly. The genome was composed of 44.61% repetitive sequences and 29,623 protein-coding genes, 97.62% of protein-coding genes could be functionally annotated. Phylogenetic and chromosome syntenic analysis revealed that *F. pentaphylla*-FP039 was closely related to *F. nubicola*. This high-quality genome could provides fundamental molecular resources for evolutionary studies, breeding efforts, and exploring the unique biological characteristics of *F. pentaphylla*.

## Introduction

Cultivated strawberry (*Fragaria* × *ananassa*) is the most widely cultivated fruit crop in the world, which is an allo-octoploid species originating nearly 300 years ago form wild progenitors form the Americas. Currently, there are 24 wild species of the genus *Fragaria* (Rosaceae), with various ploidies and mainly distributing in America, Asia, and Eurasia (Staudt et al., 2003; Liu and Davis, 2011; Liston et al., 2014). Each species has its own characteristics, *F. vesca*, the most widely distributed and the earliest domesticated wild species, is a model plant for the Rosaceae family (Darrow, 1966; Shulaev et al., 2008; Alger et al., 2018). To elevate the model plant system for more efficient application, the genome sequencing, phylogenetic evolution and functional analysis of strawberry have remained the hot research topics. The first reference genome of *Fragaria* was published in 2011, sequenced an accession of *F. vesca* named “Hawaii-4”, then multiple versions of upgrades and annotations have been released successively (Shulaev et al., 2011; Tennessen et al., 2014; Edger et al., 2018; Li et al., 2019). Recently, a chromosome-scale genome of another *F. vesca* accession “CFRA 2339”, produces red fruit, flowers perpetually, and runnerless was available online, which will serve as a valuable new resource by expanding the phenotypic traits (Alger et al., 2021). Following *F. vesca*, genomes of *F. × ananassa* (Edger et al., 2019), *F. iinumae* (Edger et al., 2020), *F. nilgerrensis* (Zhang et al., 2020), *F. viridis* (Feng et al., 2021), *F. daltoniana* (Qiao et al., 2021), *F. pentaphylla* (Qiao et al., 2021), *F. mandshurica* (Qiao et al., 2021), and *F. nubicola* (Feng et al., 2021) were also assembled to date. All these sequences not only provided opportunity to further understand the genomic features and phylogenetic relationships, but also gave us more questions because of the lack of information about the whole genus. So more high-quality genomes of new genus members and different accessions were needed.

*Fragaria pentaphylla*, a diploid species of *Fragaria* (2*n* = 2 × = 14), is endemic to Southwest China, mainly distributed in Sichuan, Gansu, and Shaanxi Province etc. (Lei et al., 2017). Same as *F. vesca*, it has two morphs of red and white fruit color (Chen et al., 2020). In terms of fragrance and resistance, *F. pentaphylla* also has unique features. Compared to cultivated strawberry, berries of *F. pentaphylla* have more varieties of volatile compounds, and the high level of the predominant volatiles 3 (2H)-furanone 4-methoxy-2,5 methyl led to a stronger aroma of white-fruited types, besides, *F. pentaphylla* has higher levels of aromatic compound methyl anthranilate than *F. vesca* (Ulrich et al., 1997; Duan et al., 2018). Resistance screening studies suggested that *F. pentaphylla* expressed highly resistant to *Xanthomonas fragariae* and moderately resistant to *Phytophthora cactorum* (Xue et al., 2005; Eikemo et al., 2010). Meanwhile, our previous study showed the whole *Fragaria* could be clustered into two clades based on molecular phylogenetic analysis together with growth characteristics and geography distribution. *F. pentaphylla* is a representative species in the South Clade, the divergence of this clade was relatively late, at around 0.63 Mya except the most ancient species *F. iinumae*, which diverged at around 3.44 Mya. Of the diploid species, *F. pentaphylla* and *F. nubicola* were the only two typical quinquefoliolate species, and this character was only observed in the south clade species (Sun et al., 2021). All these results indicated that *F. pentaphylla* is not only a valuable breeding material, but also an important species for studying the origin and evolution of *Fragaria*. Furthermore, *F. pentaphylla* has the potential to become another model plant for research due to its smaller genome and characteristic agronomic traits.

In this study, we aim to assemble a high-quality chromosome-level reference genome of a *F. pentaphylla*accession, BAAFS-FP039, using PacBio single molecule real-time (SMRT) sequencing and high-throughput chromosome conformation capture (Hi-C) technologies. The resolve of this genome would give new insight into the evolution of the genus *Fragaria*, and provide more information for further molecular biology studies.

## Data

### Genome sequencing and assembly

**FIGURE 1:**
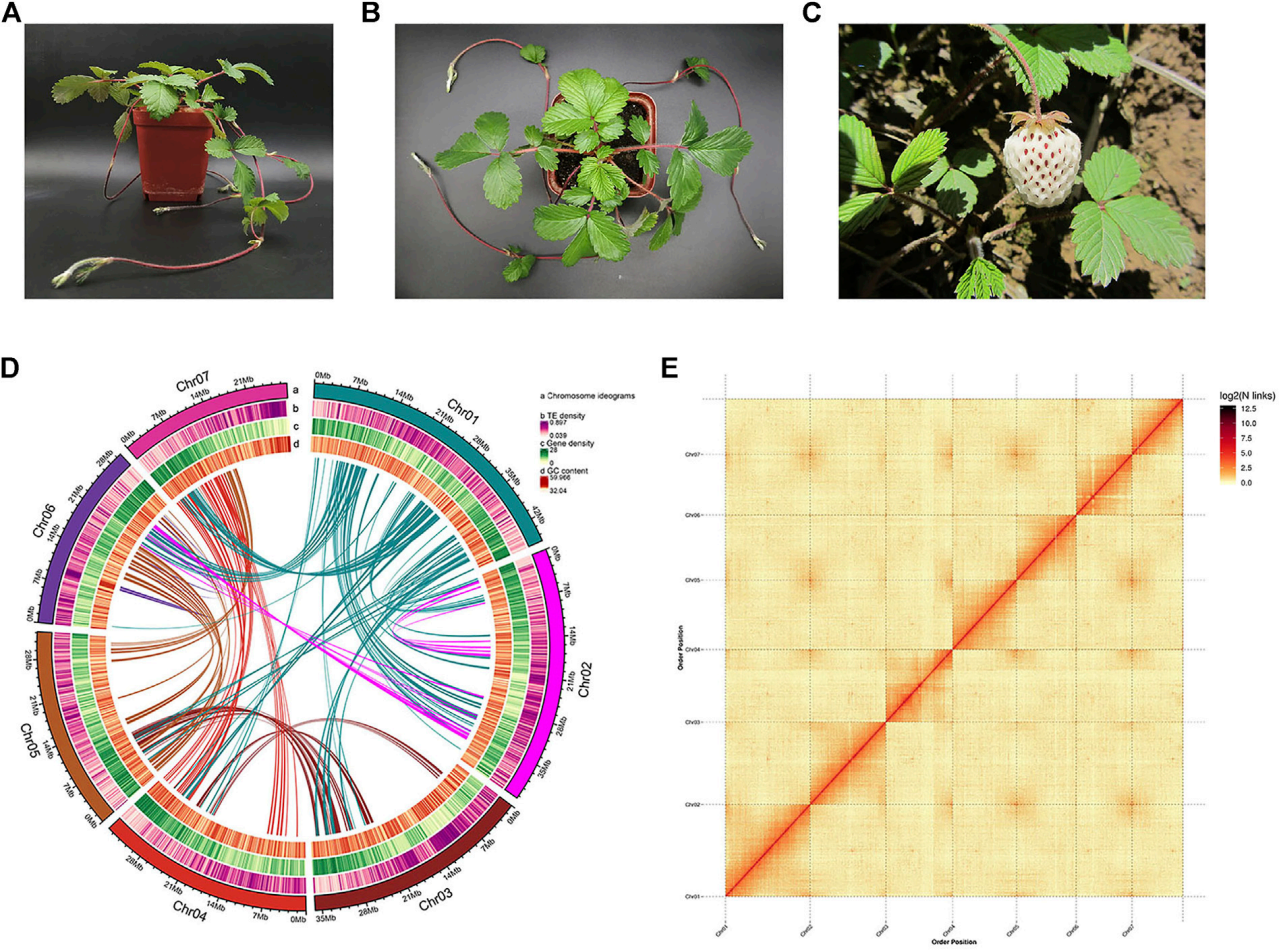
Genome assembly of *Fragaria pentaphylla*-FP039. **(A,B)**: Pictures of *Fragaria pentaphylla*-FP039 plant grown in the laboratory. **(C)** Pictures of *Fragaria pentaphylla*-FP039 plant grown in the wild where it was collected. **(D)** Circos plot of chromosome-level scaffolds showing the genomic features. Tracks from outside to the inner correspond to a, chromosome ideograms; b, TE (transposable elements) density; c, gene density; d, GC content. **(E)** Hi-C interaction heatmap showing interactions among seven chromosomes (the color bar on the right showing the interaction intensity).

A single plant of *Fragaria pentaphylla*-FP039 with white fruit was used for genome sequencing. Then we obtained 15.15 Gb Illumina short reads (79.69-fold-coverage), 33.05 Gb PacBio SMRT reads (128.73-fold-coverage), and 33.47 Gb Hi-C data (131.19-fold-coverage) (Table 1). Based on the K-mer analysis, the estimated genome size was 256.77 Mb, and the genome heterozygosity rate, proportion of repeat sequences and GC content were 1.00%, 44.61%, and 39.69%, respectively. The high accuracy CCS (Circular Consensus Sequencing) data were assembled into 41 contigs with a total length of 256.74 Mb and a contig N50 length of 41.06 Mb. Subsequently, based on the Hi-C clean data, CCS data were corrected and scaffolded into seven pseudo-chromosomes with a total length of 255.17 Mb, a contig and scaffold N50 length of 32.38 Mb and 34.60 Mb, respectively, (99.39% of the total length) (Table 1 and Figure 1D). 3D-DNA analysis was carried out to help Hi-C assembly, the generated parameters were showed in Supplementary Table S1. In conclusion, 97.11% scaffolded sequences could determine the sequence and direction.

**TABLE 1:** Summary statistics of the sequencing and assembly of the *Fragaria pentaphylla-*FP039 genome.

Library type	Sequencing mode	Clean data (Gb)	Application
Illumina	Pair end 150 bp	15.15	Genome survey and correction
PacBio	Sequel II HiFi	33.05	Genome assembly
Hi-C	Pair end 150 bp	33.47	Assisted assembly at the chromosomal level
Genome assembly and scaffolding at chromosomal level			
Contig number		44	
Contig length (bp)		256,736,466	
Contig N50 (bp)		32,376,794	
Contig N90 (bp)		11,400,560	
Scaffold number		41	
Scaffold length (bp)		256,736,766	
Scaffold N50 (bp)		34,602,923	
Scaffold N90 (bp)		27,601,762	
Anchored chromosomes size (bp)		255,168,039	

### Evaluation of the genome assembly

The quality of the assembled genome was evaluated using multiple approaches. The consensus quality (QV), error rate and K-mer completeness were estimate as 54.76, 3.34431e-06, and 84.71% (Supplementary Figure S1), respectively, using Mercury version 1.3, which indicated the high accuracy and completeness of the assembled genome at base-level. The Illumina and PacBio reads were aligned to the genome, and the re-mapping rate were 97.46% and 99.76%, respectively, (Supplementary Table S2). BUSCO analysis showed that the assembled genome contained 1,594 (98.76%) complete BUSCOs, including 1,558 single copy BUSCOs, and 36 duplicated BUSCOs (Supplementary Table S3). CEGMA analysis showed that the assembled genome completely covered 457 (99.78%) of the 458 CEGs (core eukaryotic genes), and 242 (97.58%) of the 248 highly conserved CEGs (Supplementary Table S4). The LTR Assembly Index (LAI) score was 21.27, which reached the Gold level. The Hi-C heatmap demonstrated that interactions within the chromosome were stronger than the inter-chromosomal interactions (Figure 1E). These results indicated that the genome assembly had high quality and completeness.

### Annotation of the genome assembly

After genome assembly, a total of 29,623 genes were predicted (Supplementary Table S5). The average gene length was 3,179.35 bp, the mean exon number of each gene was 5.21, and the average coding sequence length was 1,445.77 bp (Supplementary Table S6). Among these genes, 97.62% could be functionally annotated based on NR, GO, and KEGG databases (Supplementary Table S7). The annotation of the noncoding RNA genes yielded 750 tRNA, 653 rRNA, 52 miRNA, 119 snRNA, and 381 snoRNA (Supplementary Table S8). A total of 184,090 transposable elements and 128,776 tandem repeats were predicted (Supplementary Tables S9, S10). In addition, 232 pseudogenes were predicted, with an average length of 4,697.59 bp (Supplementary Table S11). These results provide a valuable genetic resource for future functional genomics.

### Evolution analysis

**FIGURE 2:**
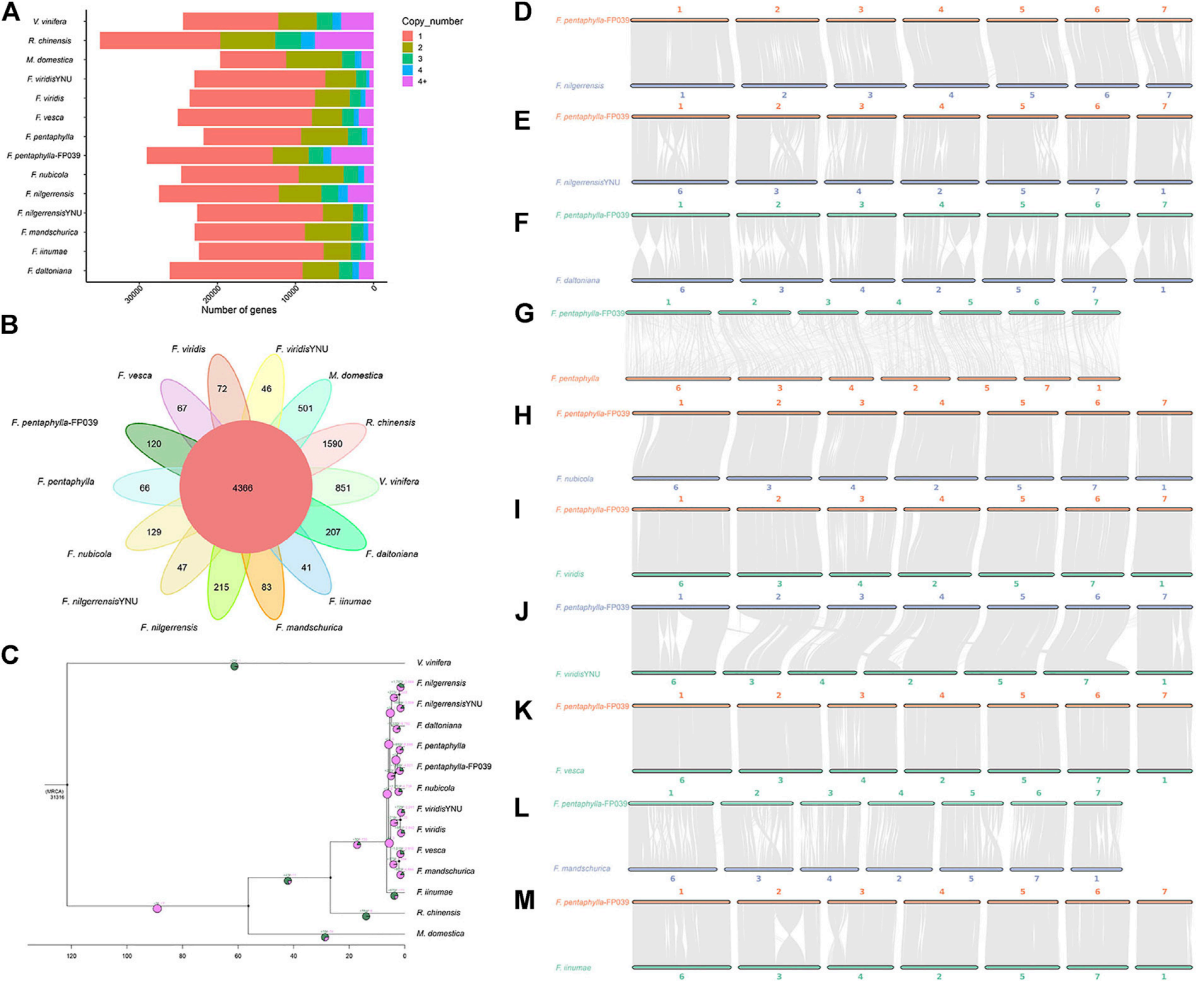
Comparative genomics analysis of *Fragaria pentaphylla*-FP039 genome and other representative plant genomes. **(A)**: Distribution of genes with different copy number in analyzed genomes. **(B)**: Venn diagram of the shared and unique orthogroups calculated among the fourteen genomes. The number of shared orthogroups was showed in the center. And numbers of unique orthogroups of corresponding species were indicated outside. **(C)**: Phylogenetic tree of 14 plant genomes with numbers of expanded (+)/contracted (−) orthogroups at each branch. The ratio of expansion and contraction orthogroups are indicated in the pie diagram with green and purple color, respectively. **(D–M)**: Synteny analysis between *F. pentaphylla*-FP039 genome and the other ten genomes of diploid *Fragaria* species.

Through the structural and functional annotation analysis of orthogroups, 35,351 orthogroups were clustered in fourteen genomes of eleven species, including *Vitis vinifera*, *Malus domestica*, *Rosa chinensis*, *Fragaria iinumae*, *Fragaria viridis*, *Fragaria nilgerrensis*, *Fragaria nubicola*, *Fragaria vesca*, *Fragaria daltoniana*, *Fragaria mandshurica*, and *Fragaria pentaphylla* (Supplementary Table S12). Among these 4,366 common orthogroups, and 120 orthogroups were specific to *Fragaria pentaphylla-*FP039 (Figures 2A, B and Supplementary Table S13). KEGG (Kyoto Encyclopedia of Genes and Genomes) enrichment analysis indicated that these specific orthogroups were significantly enriched in amino sugar and nucleotide sugar metabolism, alanine, aspartate and glutamate metabolism, diterpenoid biosynthesis, RNA polymerase, and ascorbate and aldarate metabolism (Supplementary Figure S2, Supplementary Table S14). A total of 1,003 single-copy genes were used to construct a species phylogenetic tree. The phylogenetic relationships showed that the calculated *Fragaria* species mainly divided into two clades shared *F. iinumae* as the same ancient species, which was consistent with the results of Qiao et al. (2021). Also, the different accessions of *F. pentaphylla*, *F. nilgerrensis*, and *F. virids* clustered together, respectively (Figure 2C). The synteny analysis conducted between the *F. pentaphylla*-FP039 genome versus the other ten genomes of diploid *Fragaria* species indicated that *F. pentaphylla*-FP039 had more conserved syntenic relationships with *F. nubicola*. However, we were hard to detect long syntelogs between the genomes of two accessions of *F. pentaphylla* due to the overall fragmented assembly of the genome released by Qiao et al. (2021) with a contig N50 length of 0.91 Mb (Figure 2D–M). Subsequently, the gene family expansion and contraction within the fourteen genomes were investigated. In total, 1,496 and 4921 orthogroups were expanded and contracted in *Fragaria pentaphylla*-FP039 compared with the other thirteen genomes, respectively (Figure 2C). KEGG enrichment analysis indicated that expanded genes were enriched in sesquiterpenoid and triterpenoid biosynthesis, RNA polymerase, and photosynthesis (Supplementary Figure S3, Supplementary Table S15), whereas contracted genes were enriched in flavonoid biosynthesis, oxidative phosphorylation, and stilbenoid, diarylheptanoid and gingerol biosynthesis (Supplementary Figure S4, Supplementary Table S16).

## Materials and methods

### Sample collection

The *Fragaria pentaphylla* plants used for genome sequencing were collected from Guangyuan, Sichuan province (105°59′08″E, 32°38′38″N, 1348 m) in 2016. Then it was preserved in the open field in China National Strawberry Germplasm Repository (Beijing, China) with the number BAAFS-FP039 and propagated by runners every year. This accession was characterized by larger white fruits and intense fruit aroma (Figures 1A–C). Young leaves of a single plant were used to extract genomic DNA by a modified CTAB (cetyl trimethyl ammonium bromide) method as Chang described (Chang et al., 2007). The quality and quantity of DNA were separately assessed using electrophoresis on agarose gel and a Spectrophotometer (DeNovix, United States).

### Genome features estimation from K-mer method

A 350 bp short-insert library was constructed in accordance with Illumina’s instructions (San Diego, CA, United States). Then the short-reads (pair end 150 bp) from Illumina platform were quality filtered, and generated 15.15 Gb high-quality clean reads. The high-quality clean reads were used for genome size estimation by conducting k-mer (k = 19) analysis (Marcais and Kingsford, 2011; Ranallo-Benavidez et al., 2020).

### Libraries construction and PacBio, Hi-C sequencing

For genome sequencing, we constructed a long-insert library following PacBio Sequel’s instruction. Genomic DNA was sheared into ∼15 kb fragments by g-TUBE, the SMRTbell library was constructed using the SMRTbell Express Template Prep kit 2.0 (Pacific Biosciences). After DNA damage repair, end repair, ligation with T-overhang, exonuclease digestion size selection, and library purification, the size and quality of the library were assessed. Finally, the PacBio Sequel II platform (PacBio Biosciences, Menlo Park, CA, United States) were employed for whole-genome sequencing according to the standard protocols.

The Hi-C (high-throughput chromosome conformation capture) sequencing was performed to construct the chromosome-level assembly of *Fragaria pentaphylla*. The Hi-C fragment library was constructed as Rao et al. (2015) described, the fresh sample was fixed with formaldehyde, then the cross-linked DNA was digested with restriction enzyme *HindIII*, repaired with biotinylated residues, ligation with T4 DNA ligation enzyme, and reverse-crosslinked. Next, the DNA was purified and sheared to fragments of 300–700 bp to construct the Hi-C library, and the final library was sequenced through the Illumina HiSeq X Ten platform (Illumina Inc., San Diego, CA, United States) with the PE150 sequencing strategy.

### Genome assembly based on PacBio and Hi-C data, and quality assessment

For genome assembly, the raw reads generated from the PacBio platform were filtered, then high accuracy CCS data were assembled using hifiasm (version 0.12) software (Cheng et al., 2021) to obtain genome sequences. For chromosome-level assembly, the adapter sequences of raw reads and low-quality PE reads were removed. Then invalid read pairs were filtered by HiC-Pro v2.10.0 (Servant et al., 2015). LACHESIS software (Burton et al., 2013) and Juicer version 1.6 (Durand et al., 2016) were used for chromosome-level scaffolds. Before chromosomes assembly, preassembly for error correction of scaffolds was performed by BWA (version 0.7.10-r789) software (Li and Durbin, 2009). To evaluate the quality of genome assembly, the Illumina and PacBio reads were aligned to the genome using BWA and minimap2 version 2.24-r1122 (Li, 2018), and the BUSCO (Benchmarking Universal Single-Copy Orthologs) version 4.0 (Waterhouse et al., 2018) and CEGMA (Core Eukaryotic Genes Mapping Approach) (Dong et al., 2020) were used to assess the integrity of genome assembly. Merqury was hired to evaluate the base-level accuracy and completeness (Rhie et al., 2020). LAI (LTR Assembly Index) score was also used to assess genome assembly quality (Ou et al., 2018).

### Genome annotation

TEs (Transposon elements) were identified through homology-based and de novo-based strategies. We first customized a *de novo* repeat library and a high-quality intact fl-LTR-RTs (full-length long terminal repeat retrotransposons) and non-redundant LTR library through RepeatModeler2 (version 2.0.1) and LTR_retriever (version 2.8), respectively. Then a non-redundant species-specific TE library was constructed based on the *de novo* TE sequences library above and the known Repbase (version 19.06), REXdb (V3.0), and Dfam (v3.2) database. Finally, TE sequences in the *Fragaria pentaphylla* genome were identified and classified by homology search against the library using RepeatMasker (version 4.1.0). Tandem repeats were annotated by Tandem Repeats Finder (TRF 4.09) and MIcroSAtellite identification tool (MISA version 2.1).

Protein-coding genes were annotated through the combination of *de novo* prediction, homology search, and transcript-based assembly. For *de novo* prediction, Augustus (version 2.4) and SNAP (2006-07-28) were used to predict *de novo* gene models. GeMoMa (v1.7) software was performed for homology-based prediction, and using reference gene models from *A. thaliana*, *F. nilgerrensis*, *F. vesca*, *R. chinensis*, and *V. vinifera*. For transcript-based assembly, the transcriptome sequencing was performed using young leaves, stems and roots of *Fragaria pentaphylla*-FP039. The RNA-seq data were aligned to the reference genome using Hisat (version 2.0.4), then GeneMarkS-T (version 5.1), and PASA (version 2.0.2) were used to predict genes. All the prediction results were combined using the EVM software (version 1.1.1). Gene functions were inferred based on the best match of the alignments to the NCBI (National Center for Biotechnology Information), EggNOG, TrEMBL, Swiss-Prot, and KOG protein databases.

For pseudogene prediction, the GenBlastA (version 1.0.4) program was used to scan the whole genomes, then GeneWise (version 2.4.1) was used to search for non-mature mutations and frame-shift mutations.

For Non-coding RNAs annotation, the tRNAscan-SE (version 1.3.1) was used to predict tRNA, barrnap (version 0.9) was used to identify rRNA, miRBase (release 21) databases were used to identify miRNA, and INFERNAL and the Rfam (release 12.0) database were used to identify snoRNA and snRNA.

### Genome evolution analysis

The orthogroups of *F. pentaphylla*-FP039 within its closely related species and other representative plant genomes, including *Fragaria iinumae*, *Fragaria viridis*, *Fragaria nilgerrensis*, *Fragaria nubicola*, *Fragaria vesca*, *Fragaria daltoniana*, *Fragaria mandshurica*, *Fragaria pentaphylla Vitis vinifera*, *Malus domestica*, and *Rosa chinensis* (Supplementary Table S12, Supplementary Figure S5), were identified using OrthoFinder (v2.4.0) software (Emms and Kelly, 2019) with default parameter settings, and annotated using PANTHER V15 database (Mi et al., 2019). A phylogenetic tree for the 14 plant species was constructed using the IQ-TREE v1.6.11 (Nguyen et al., 2015) with the ModelFinder (Kalyaanamoorthy et al., 2017) model based on single-copy genes. The gene synteny between the genome of *F pentaphylla*-FF039 and the genomes of other ten diploid *Fragaria* species were compared through MCScanX. Based on the identified orthogroups and the constructed phylogenetic tree, the expansion and contraction of the orthogroups were analyzed with CAFÉ v4.2 (computational analysis of gene family evolution).

## Data Availability

Our sequencing data is available in the NCBI SRA database under the BioProject PRJNA801713 and PRJNA804380, and the genome assembly and annotation information is available at Figshare (DOI: 10.6084/m9.figshare.19092221).

## Supplementary material

Supplementary Figure S1 Spectrum plots for K-mer analysis estimated by Merqury. **A**: Copy number spectrum plot for the genome assembly. The bar plotted at zero multiplicity represents base errors in the assembly. **B**: Assembly spectrum plot for evaluating k-mer completeness. K-mers are colored by their presence in the reads and assembly.

Supplementary Figure S2 Top ten KEGG pathways from the enrichment analysis of Fragaria pentaphylla-FP039 specific orthogroups. The color of column represents the *p*-value. The complete and detail information were showed in Supplementary Table S13.

Supplementary Figure S3 Top ten KEGG pathways from the enrichment analysis of Fragaria pentaphylla-FP039 expanded orthogroups. The color of column represents the *p*-value. The complete and detail information were showed in Supplementary Table S14.

Supplementary Figure S4 Top ten KEGG pathways from the enrichment analysis of Fragaria pentaphylla-FP039 contracted orthogroups. The color of column represents the *p*-value. The complete and detail information were showed in Supplementary Table S15.

Supplementary Figure S5 Pictures of some relative species of *Fragaria pentaphylla*. A: *Fragaria pentaphylla*-red fruited type; B: *Fragaria mandshurica*; C: *Fragaria vesca*-red fruited type; D: *Fragaria vesca*-white fruited type; E: *Fragaria nilgerrensis*; F: *Fragaria nubicola*.
