# Supplementary figures and images for: Chromosome-level genome assembly of Fragaria pentaphylla using PacBio and Hi-C technologies
Source: Front Genet. 2022 Sep 6;13:873711. doi: 10.3389/fgene.2022.873711 (PMC9485601; doi:10.3389/fgene.2022.873711)

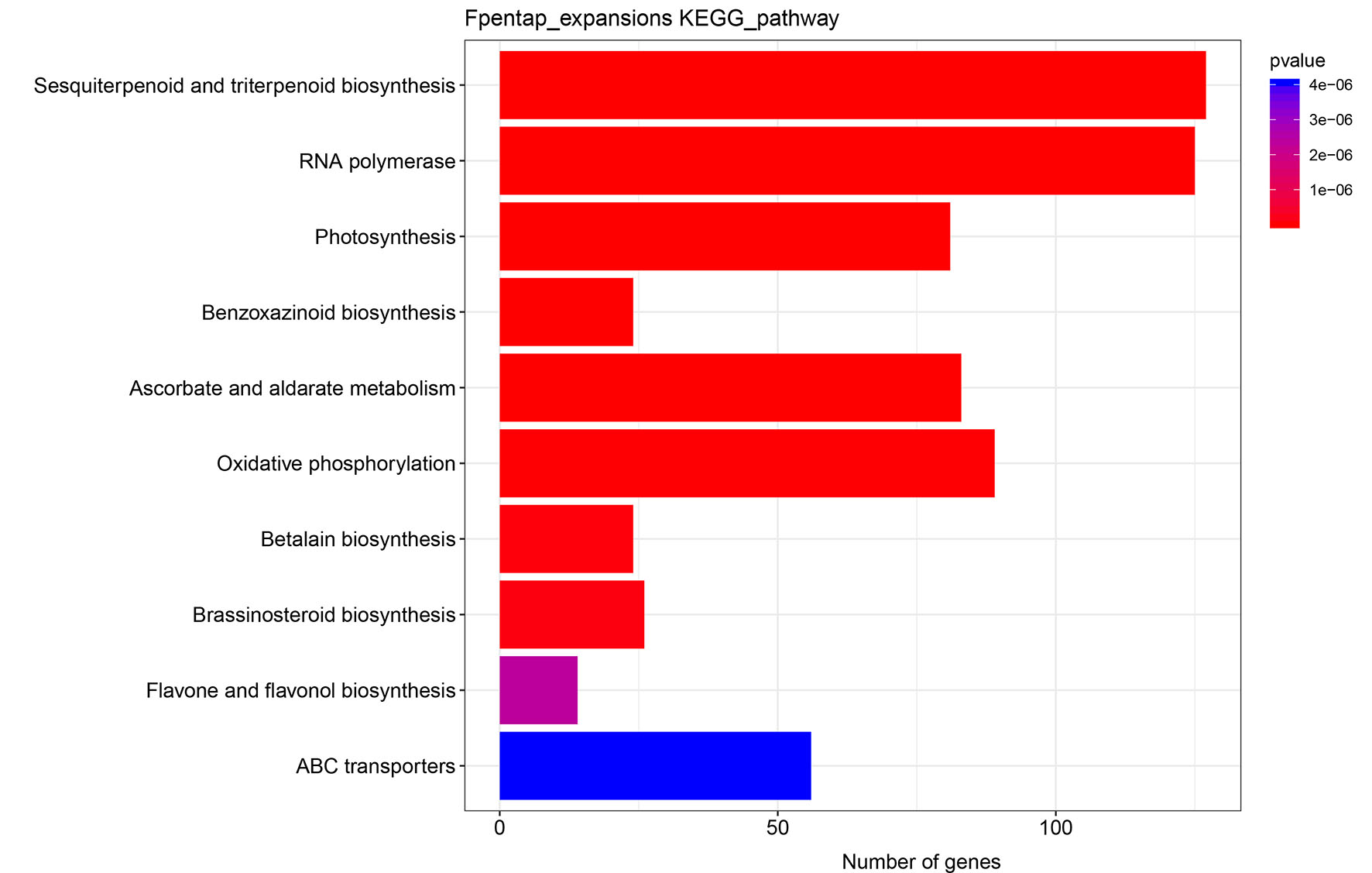

Supplement: Supplementary file 1 [file Image3.JPEG]

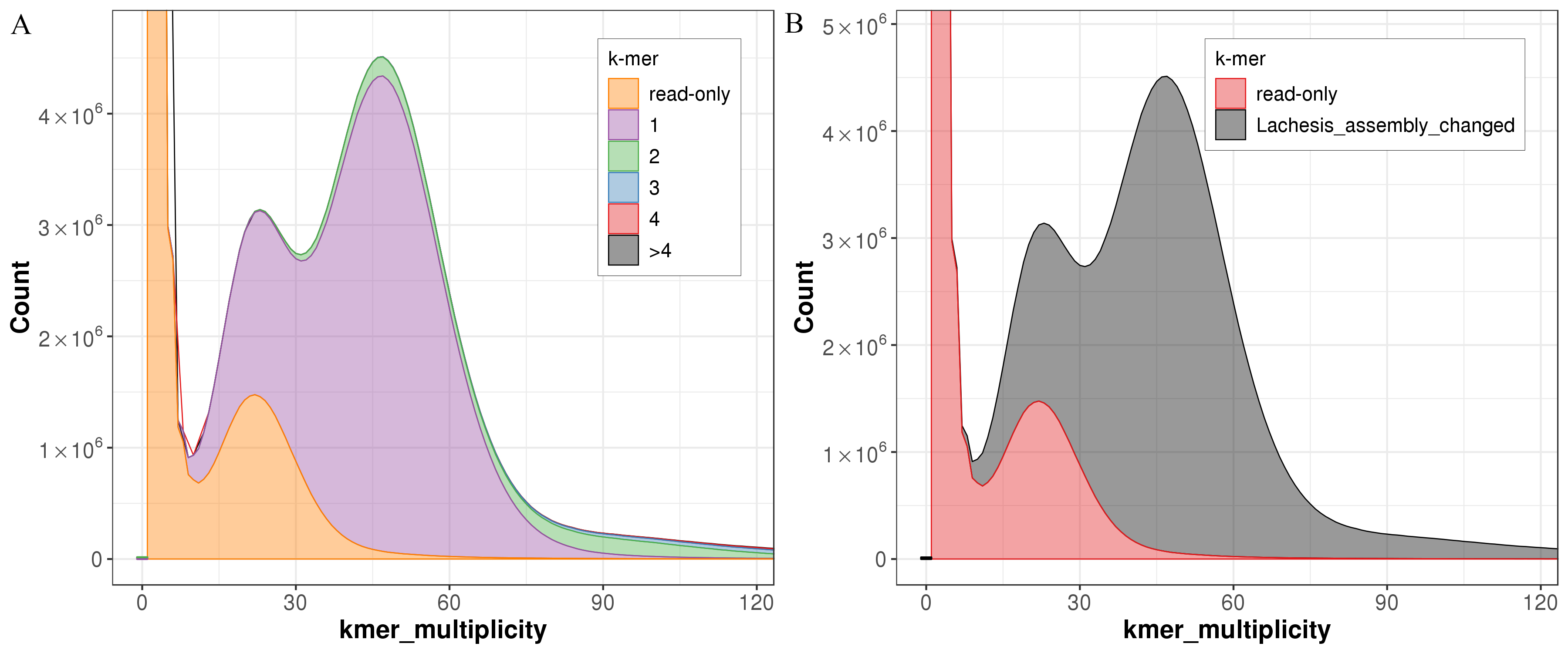

Supplement: Supplementary file 2 [file Image1.JPEG]

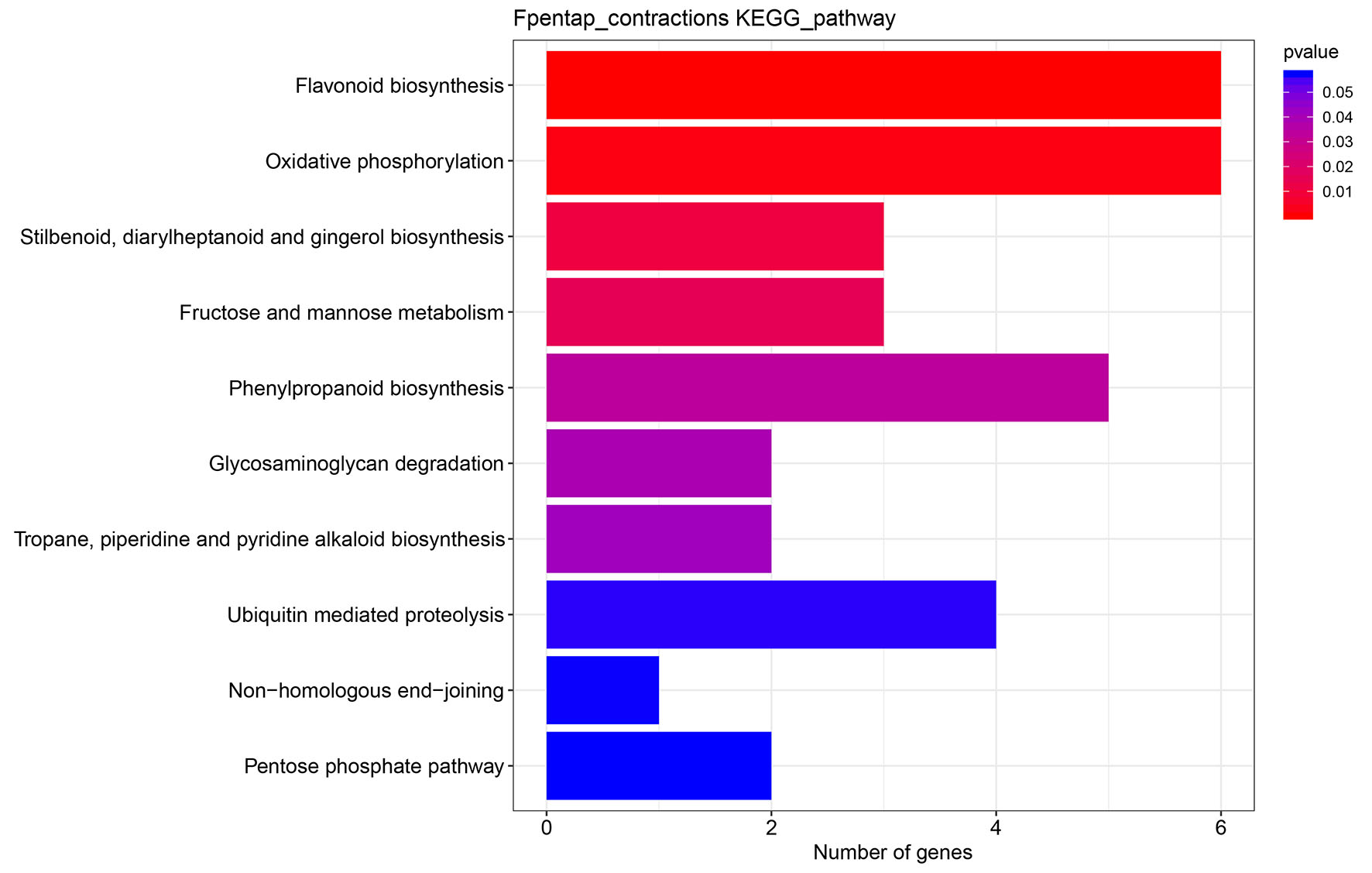

Supplement: Supplementary file 3 [file Image4.JPEG]

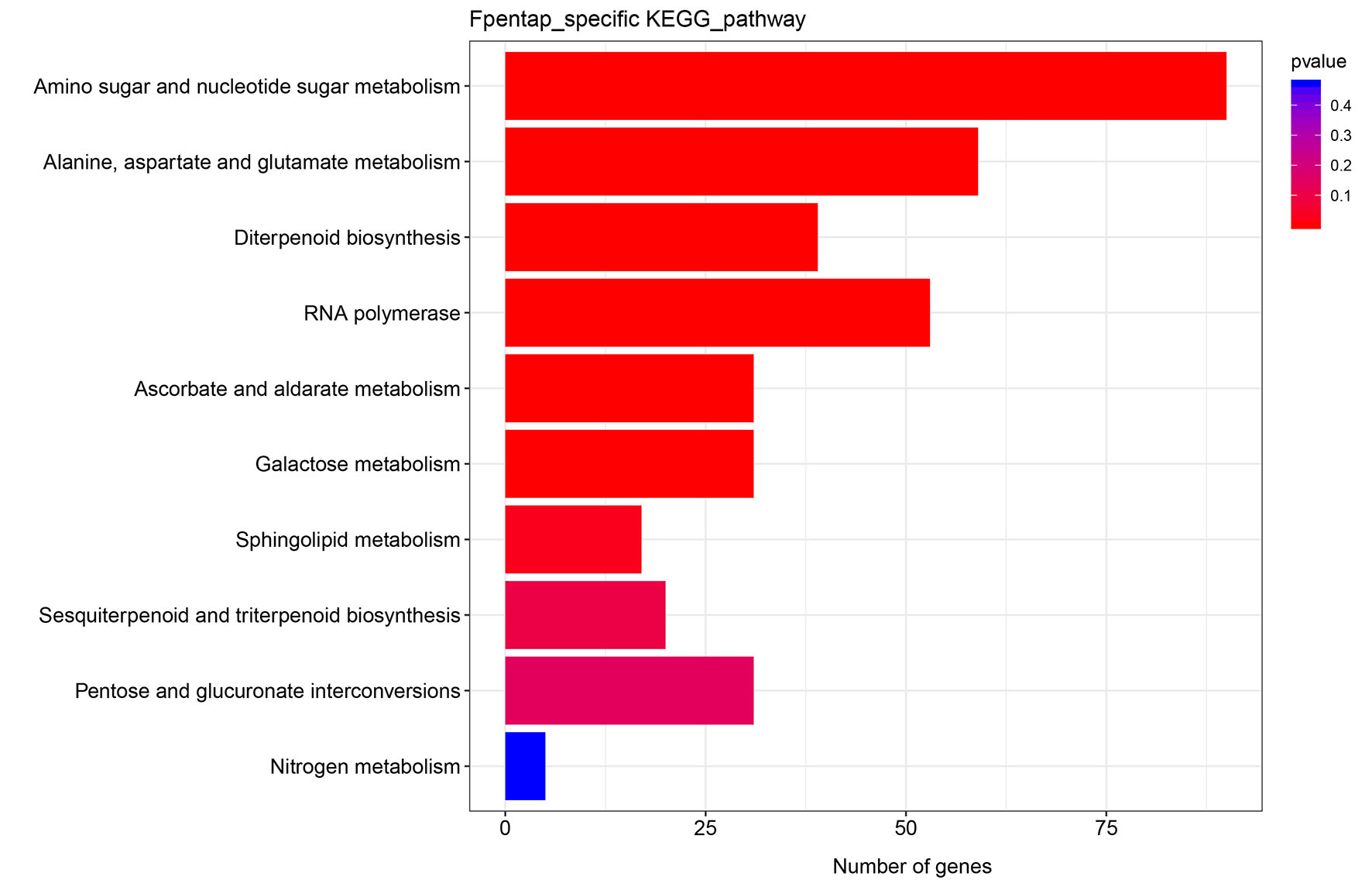

Supplement: Supplementary file 4 [file Image2.JPEG]

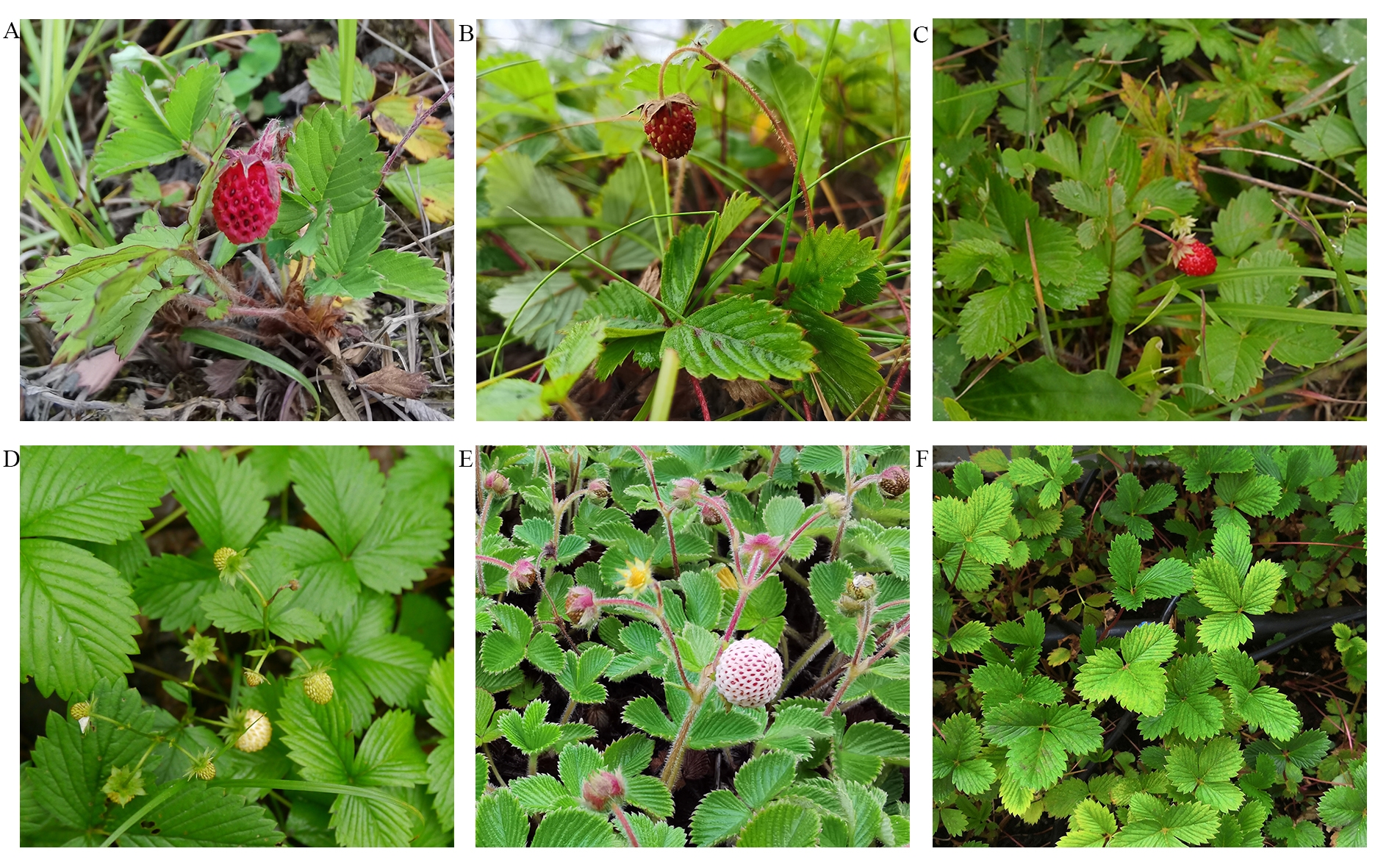

Supplement: Supplementary file 5 [file Image5.JPEG]
