# Supplementary material for: Chromosome-level genome assembly of Fragaria pentaphylla using PacBio and Hi-C technologies
Source: Front Genet. 2022 Sep 6;13:873711. doi: 10.3389/fgene.2022.873711 (PMC9485601; doi:10.3389/fgene.2022.873711)
Supplement: Supplementary file 6 [file DataSheet1.docx]

Table S1 The parameters of 3D-DNA analysis based on Hi-C reads.

| Item | Number of reads (rate of total reads) |
| --- | --- |
| Sequenced Read Pairs | 111,740,955 |
| Normal Paired | 64,274,258 (57.52%) |
| Chimeric Paired | 34,259,364 (30.66%) |
| Chimeric Ambiguous | 11,312,942 (10.12%) |
| Unmapped | 1,894,391 (1.70%) |
| Library Complexity Estimate | 85,600,302 |
| Hi-C Contacts | 34,803,037 (31.15%) |
| Inter-chromosomal Contacts | 8,917,728(7.98%) |
| Intra-chromosomal Contacts | 25,885,309(23.17%) |
| Short Range (<20Kb) | 8,656,144(7.75%) |
| Long Range (>20Kb) | 17,229,078(15.42%) |

Table S2 The consistency assessment of sequencing reads to assembled genomes.

| Library type | Total_reads | Mapped_reads | Mapped (%) | Properly_mapped_reads | Properly_mapped (%) |
| --- | --- | --- | --- | --- | --- |
| Illumina | 136,634,798 | 133,168,625 | 97.46% | 129,610,074 | 94.86% |
| PacBio | 1,865,857 | 1,861,459 | 99.76% | 0 | 0% |

Table S3 BUSCO analysis of the assembled genome.

| Complete BUSCOs(C) | Complete and single-copy BUSCOs(S) | Complete and duplicated BUSCOs(D) | Fragmented BUSCOs(F) | Missing BUSCOs(M) |
| --- | --- | --- | --- | --- |
| 1594(98.76%) | 1558(96.53%) | 36(2.23%) | 3(0.19%) | 17(1.05%) |

Table S4 CEGMA analysis of the assembled genome.

| Number of 458 CEG^*^  present in assembly | Percentage of 458 CEGs  present in assembly | Number of 248 highly conserved CEGs  present in assembly | Percentage of 248 highly conserved CEGs present in assembly |
| --- | --- | --- | --- |
| 457 | 99.78% | 242 | 97.58% |

Table S5 Statistics of predicted protein-coding gene number in *Fragaria pentaphylla*-FP039 genome.

| Method | Software | Species | Gene number |
| --- | --- | --- | --- |
| Ab initio | Augustus | - | 23,705 |
|  | SNAP | - | 41,200 |
| Homology-based | GeMoMa | *A. thaliana* | 22,704 |
|  |  | *F. nilgerrensis* | 14,754 |
|  |  | *F. vesca* | 31,917 |
|  |  | *R. chinensis* | 34,350 |
|  |  | *V. vinifera* | 23,101 |
| RNAseq | GeneMarkS-T | - | 17,224 |
|  | PASA | - | 19,891 |
| Integration | EVM | - | 29,623 |

Table S6 Analysis of predicted protein-coding genes in *Fragaria pentaphylla*-FP039 genome.

| Item | Statistics |
| --- | --- |
| Gene number | 29,623 |
| Gene length (bp) | 94,181,876 |
| Average gene length (bp) | 3179.35 |
| Exon length (bp) | 53,004,979 |
| Average exon length (bp) | 1789.32 |
| Exon number | 154,203 |
| Average exon number | 5.21 |
| CDS length (bp) | 42,827,958 |
| Average CDS length (bp) | 1445.77 |
| CDS number | 148,108 |
| Average CDS number | 5 |
| Intron length (bp) | 41,176,897 |
| Average intron length (bp) | 1390.03 |
| Intron number | 124,580 |
| Average intron number | 4.21 |

Table S7 Statistics of annotated gene number in *Fragaria pentaphylla*-FP039 genome by different databases and methods.

| Annotated database | Annotated number | Annotated ratio (%) |
| --- | --- | --- |
| GO Annotation | 23,573 | 79.58 |
| KEGG Annotation | 21,804 | 73.6 |
| KOG Annotation | 15,337 | 51.77 |
| Pfam Annotation | 25,068 | 84.62 |
| Swissprot Annotation | 22,457 | 75.81 |
| TrEMBL Annotation | 28,593 | 96.52 |
| eggNOG Annotation | 22,843 | 77.11 |
| nr Annotation | 28,877 | 97.48 |
| All Annotated | 28,918 | 97.62 |

Table S8 Statistics of non-coding RNAs in *Fragaria pentaphylla*-FP039 genome.

| rRNA number | tRNA number | miRNA number | snRNA number | snoRNA number |
| --- | --- | --- | --- | --- |
| 653 | 750 | 52 | 119 | 381 |

Table S9 Statistics of transposable elements in *Fragaria pentaphylla*-FP039 genome.

| Type | Number | Length (bp) | Rate (%) |
| --- | --- | --- | --- |
| ClassI:Retroelement | 104,572 | 64,531,587 | 25.14 |
| ClassI/DIRS | 422 | 90,221 | 0.04 |
| ClassI/LINE | 19,728 | 5,013,287 | 1.95 |
| ClassI/LTR/Cassandra | 10 | 853 | 0.00 |
| ClassI/LTR/Caulimovirus | 562 | 789,558 | 0.31 |
| ClassI/LTR/Copia | 19,680 | 16,125,654 | 6.28 |
| ClassI/LTR/ERV | 4,374 | 291,066 | 0.11 |
| ClassI/LTR/Gypsy | 22,605 | 22,215,646 | 8.65 |
| ClassI/LTR/Pao | 452 | 40,796 | 0.02 |
| ClassI/LTR/Unknown | 34,819 | 19,692,393 | 7.67 |
| ClassI/LTR/Viper | 29 | 3,674 | 0.00 |
| ClassI/SINE | 1,891 | 268,439 | 0.10 |
| ClassII:DNA transposon | 79,518 | 30,432,790 | 11.85 |
| ClassII/Academ | 21 | 1,930 | 0.00 |
| ClassII/CACTA | 14,603 | 13,706,341 | 5.34 |
| ClassII/Crypton | 518 | 31,776 | 0.01 |
| ClassII/Dada | 237 | 47,513 | 0.02 |
| ClassII/Ginger | 143 | 7,336 | 0.00 |
| ClassII/Helitron | 1,808 | 1,114,748 | 0.43 |
| ClassII/IS3EU | 108 | 4,666 | 0.00 |
| ClassII/Kolobok | 316 | 20,981 | 0.01 |
| ClassII/MITE | 33 | 4,002 | 0.00 |
| ClassII/Maverick | 326 | 16,690 | 0.01 |
| ClassII/Merlin | 69 | 2,841 | 0.00 |
| ClassII/Mutator | 4,718 | 1,279,610 | 0.50 |
| ClassII/Novosib | 263 | 11,978 | 0.00 |
| ClassII/P | 151 | 6,017 | 0.00 |
| ClassII/PIF-Harbinger | 4,468 | 1,406,748 | 0.55 |
| ClassII/PiggyBac | 256 | 14,406 | 0.01 |
| ClassII/Sola | 170 | 7,960 | 0.00 |
| ClassII/Tc1-Mariner | 2,626 | 257,347 | 0.10 |
| ClassII/Unknown | 35,162 | 9,707,032 | 3.78 |
| ClassII/Zator | 12 | 529 | 0.00 |
| ClassII/Zisupton | 259 | 14,044 | 0.01 |
| ClassII/hAT | 13,251 | 2,768,295 | 1.08 |
| Total | 184,090 | 94,964,377 | 36.99 |

Table S10. Statistic of tandem repeats in *Fragaria pentaphylla*-FP039 genome.

| Type | Number | Length (bp) | Rate (%) |
| --- | --- | --- | --- |
| Microsatellite (1-9 bp units) | 116,682 | 2,401,941 | 0.94 |
| Minisatellite (10-99 bp units) | 3,148 | 2,204,336 | 0.86 |
| Satellite (>=100 bp units) | 8,946 | 7,806,231 | 3.04 |
| Total | 128,776 | 12,412,508 | 4.83 |

Table S11 Statistic of pseudogenes in *Fragaria pentaphylla*-FP039 genome.

| Pseudogene | Statistics |
| --- | --- |
| Total Number | 232 |
| Total length | 1,089,841 |
| Average length | 4697.59 |

Table S12 Websites of published genomes using for comparative genomic analysis.

| Species | Website |
| --- | --- |
| *F. viridis* | https://www.rosaceae.org/Analysis/9155217 |
| *F. daltoniana* | https://www.rosaceae.org/Analysis/11885161 |
| *F. viridisYNU* | https://www.rosaceae.org/Analysis/12137895 |
| *M. domestica* | https://www.rosaceae.org/species/malus/malus_x_domestica/genome_v1.0 |
| *V. vinifera* | https://genome.jgi.doe.gov/portal/pages/dynamicOrganismDownload.jsf?organism=Vvinifera |
| *F. pentaphylla* | https://www.rosaceae.org/Analysis/12137892 |
| *R. chinensis* | https://lipm-browsers.toulouse.inra.fr/pub/RchiOBHm-V2/ |
| *F. nilgerrensisYNU* | https://www.rosaceae.org/Analysis/12137894 |
| *F. nubicola* | https://www.rosaceae.org/Analysis/9155216 |
| *F. nilgerrensis* | https://www.rosaceae.org/bio_data/8666460 |
| *F. iinumae* | https://www.rosaceae.org/species/fragaria_iinumae/genome_v1.0 |
| *F. vesca* | https://www.rosaceae.org/species/fragaria_vesca/genome_v4.0.a1 |
| *F. mandschurica* | https://www.rosaceae.org/Analysis/12137893 |
